# Experimental study on the mechanism of ice layer formation in fractured rock masses in cold regions

**DOI:** 10.1038/s41598-022-22735-7

**Published:** 2022-10-26

**Authors:** Liping Wang, Zhuxu Dong, Ning Li, Wenli Wang, Yanzhe Tian, Shuanhai Xu

**Affiliations:** 1 School of Civil Engineering and Architecture, Xi’an University of Technology, Xi’an, 710048 China; 2 Institute of Engineering Geology, CCTEG XI’AN Research Institute, Xi’an, 710077 China

**Keywords:** Environmental sciences, Natural hazards, Solid Earth sciences

## Abstract

The formation and growth of ice layers in fractured rock masses is one of the main causes of frost weathering in bedrock in cold regions. The mechanism of ice layer formation in fractured rock masses was simulated in this study by conducting frost weathering tests on a simulated rock mass with a vertical through fracture, which was fabricated by splicing two cement blocks. The test results were used to propose a novel mechanism for ice layer formation in terms of the initial crack-frost effect in which moisture mainly migrates in vapor form. The continuous accumulation and growth of these frosts eventually forms an ice layer that expands the crack, and the fractured rock simulation model undergoes frost-heaving damage. The frost layer on the rock wall in the negative temperature region is similar to the frosting process on the cold surface of thermodynamic studies. The relative humidity (RH) gradient and temperature gradient were shown to be the factors affecting water vapor migration, where the former plays a direct and dominant role, and the temperature gradient has a very limited direct effect on vapor migration.

## Introduction

Frost weathering is a fundamental geomorphic process operating from Polar region to tropical high mountains, wherever rocks experience a temperature fluctuation across the freezing point and moisture is present (Matsuoka and Murton^1^). Frost weathering consists of two physical processes, freezing and melting, in which the formation and growth of ice layers during freezing leads to cracking of bedrock or expansion of existing fissures, making the rock mass develop toward fragmentation. On the one hand, this may greatly increase bedrock’s permeability to groundwater recharge, drinking water supply and contaminant transport, and on the other hand, present significant safety hazards to the construction of projects on bedrock of cold regions. The formation and development process of these ice layers are key to understanding the frost weathering process and bedrock characteristics in permafrost areas. These results can be used to predict the long-term evolution of bedrock in cold regions and guide the design, construction and maintenance of related projects.

Two mechanisms have been proposed, in literature, for the formation of an ice layer in rock. One mechanism is in situ volumetric expansion (Matsuoka and Murton ^1^): if water completely fills the spaces in rock and freezes in situ, ice growth at – 22 °C can, in principle, generate pressures up to 207 MPa inside cracks in rock (Tsytovich^2^) that is strong enough to fracture any rock, because the maximum tensile strength of rock is one to two orders of magnitude lower than 207 MPa. However, precisely defined conditions are required for frost weathering by volumetric expansion: the rock must be nearly or completely water-saturated and freeze rapidly from all sides. Another mechanism is ice segregation: Walder and Hallet^3,4^ developed a mathematical model (W–H Model) for the breakdown of porous rock via the growth of ice lenses within cracks. Laboratory experiments have demonstrated that ice segregation can fracture porous, moist rock (Akagawa and Fukuda^5^; Hallet et al.^6^; Murton et al.^7–10^). Ice segregation does not require specific environmental conditions for in situ volumetric expansion, and the applicability of this theory to porous rocks has been demonstrated by field investigations (Büdel^11^; French et al.^12^; Hodgson et al.^13^; Mackay^14^).

Most of the existing bedrock frost weathering studies discussed above only consider the solid rock matrix, while the actual bedrock consists of both rock matrix and fractures, where fractures are often the main water storage and transport channels. As such, it is difficult to directly explain the relevant ice formation and growth process by the existing in situ volumetric expansion or ice segregation mechanism. The authors used two cement blocks spliced into a fractured rock simulation model with a vertical through fracture, and conducted a unidirectional freezing test with constant water level at the bottom of this model. Based on the test results, the main patterns and pathways of water migration are analyzed, and a new mechanism of ice formation in fractured rock masses, an initial crack-frost effect, is proposed.

## Theoretical foundation

### Vapor migration

Assume that the water vapor in the medium is in steady flow. The fundamental driving force for vapor transport is the chemical potential of the water vapor, which is typically expressed in terms of the vapor concentration or density. Variations in the temperature and vapor pressure create gradients in the vapor density that drive vapor flow. Therefore, gradients in the temperature and vapor pressure (relative humidity) are the two major driving forces for vapor transport in a medium.

The diffusive vapor flux ${\mathrm{q}}_{V}$(in kg/m^2^·s) in the medium caused by temperature and vapor pressure (relative humidity) gradients is (Lu and Likos^15^)

1 $${\mathrm{q}}_{V}=-{D}_{V}{\rho }_{v,sat}\left(\frac{\nabla RH}{RH}-\frac{\nabla \mathrm{T}}{T}+\frac{\lambda {\omega }_{w}\nabla T}{R{T}^{2}}\right),$$

where ${D}_{V}$ (m^2^/s) is the diffusion coefficient for water vapor transport in the medium, ${\rho }_{v,sat}$ (g/m^3^) is the saturated vapor density, RH (%) is the relative humidity, T (K) is the temperature, $\lambda$ is the latent heat of evaporation of water, ${\omega }_{w}$ (g/mol) is the molar mass of water vapor, and R is the gas constant (8.31 J/(mol·K)).

The negative signs of the first and third terms on the right-hand side of Eq. (1) show that vapor flows from regions with high relative humidity to those with low relative humidity and from high-temperature regions to low-temperature regions. The positive second term corresponds to a counteracting flux from low to high temperature. This offsetting term reflects the contraction of air under low temperature, which increases the vapor density, whereas air expansion under high temperature decreases the vapor density.

The second term on the right-hand side of Eq. (1) is negligible relative to the first and third terms. Then the vapor flux caused by the temperature gradient is

2 $$-{D}_{V}{\rho }_{v,sat}\frac{L{\omega }_{w}\nabla T}{R{T}^{2}}.$$

### Experimental study of ice segregation and liquid water migration of rock

Ice segregation occurs when temperature gradient-induced suction in freezing or frozen ground (or rock) drives unfrozen water through a porous media towards a freezing site, where lenses or layers of ice grow. Some typical ice segregation experiments and phenomenon is listed in Table 1.

**Table 1:** Some typical ice segregation experiments and phenomenon.

Literature	Rocks and properties	Test condition	Phenomenon
Hallet et al.^6^	Saturated Berea Sandstone (Length × width × height = 5 × 5 × 30 cm) Hydraulic conductivity: 10^-6^ m/s Tensile strength: 3 ~ 6 MPa Freezing temperature: − 0.2 °C	Unidirectional freezing test Lower constant head boundary	The signal for specimen cracking occurred mainly in the range of − 3 to − 6 °C
Akagawa and Fukuda^5^	Saturated Welded tuff (Length × width × height = 29 × 29 × 25 cm) Porosity: 37.9% Compressive strength: 14.6 MPa Tensile strength: 6.6 MPa	Unidirectional freezing test Lower constant head boundary	The segregated ice layer appeared at 7 cm from the bottom, corresponding to a temperature range of − 1.4 to − 1.5 °C It can be divided into 3 stages: in situ freezing (20 h), conversion stage (20 ~ 65 h) and segregated ice stage (> 60 h)
Murton et al.^8^	Siliceous chalk (Length × width × height = 30 × 31 × 33 cm) Porosity: 47.2% Permeability: 114.5 ~ 333millidarcies Compressive strength: 0.53 ~ 9.24 MPa Tensile strength: 0.07 ~ 1.07 MPa	Bidirectional freezing test Lasted about one year	The segregated ice layer appeared in the middle of the specimen, and only approximately 1.4 mm of frost heave appeared on the sample surface on the 28th day of the test

### Frost mechanism on cold surface

**Figure 1:**
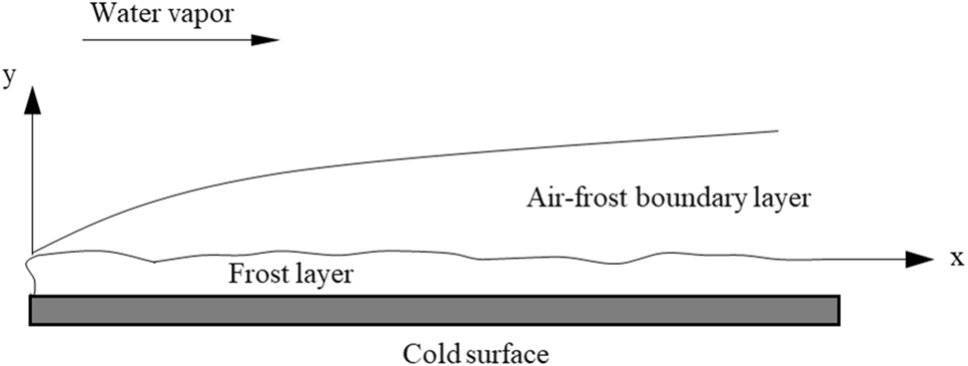
Physical model of frost growth on cold plate.

The frosting process on the negative temperature region of a single rock wall is equivalent to the frosting process on the cold surface of thermodynamic studies. The growth of the frost layer at a general cold surface consists of a formation period (Stage I), growth (Stage II) and stable growth (Stage III). The frost layer can be considered to be a porous medium during the frost growth period (Stage II) (Hayashi et al.^16^; Tao et al.^17^), where the diffusion of water vapor leads to thickening and densification of the frost layer. Figure 1 shows the corresponding physical model. Air at a temperature ${t}_{a}$ and water vapor flow over a cold surface with a temperature ${t}_{s}$. Frost forms if ${t}_{s}$ is below the freezing point. The frost surface temperature, ${t}_{p}$, is initially the same as that of the plate surface, ${t}_{s}$. As the frost layer thickens, ${t}_{p}$ increases to a value between ${t}_{s}$ and ${t}_{a}$. As ${t}_{p}$ increases, the increase in the humidity ratio of air in the neighborhood of the frost-air interface, ${W}_{p}$, decreases the driving force of the water vapor concentration gradient and consequently, the frost deposition rate. In general, the transferred water vapor partially diffuses into the porous frost layer, thus increasing the frost density, and partially deposits on the upper layers, thus increasing the frost thickness. The governing equations for the process are presented below (Tao et al.^17^).

#### Energy conservation

The total heat, Q, through the frost layer is the sum of contributions from convection and phase change:

3 $$\mathrm{Q}={\int }_{x=0}^{l}w[{h}_{x}\left({t}_{a}-{t}_{px}\right)+L{h}_{vx}({W}_{a}-{W}_{px})]dx,$$

where $w(\mathrm{m})$ is the plate width; ${h}_{x}\; (\mathrm{W}/{\mathrm{m}}^{2}\mathrm{K})$ is the local film heat transfer coefficient; ${h}_{vx}$ (kg/m^2^s) is the local convective mass transfer coefficient; ${t}_{a}$ (°C) is dry-bulb temperature of air; ${t}_{px}$ (°C) is the local frost surface temperature; $L$ (kJ/kg) is the latent heat of sublimation of frost; ${W}_{a}$ (kg_v_/kg_dry air_) is the humidity ratio of air evaluated at ${t}_{a}$; ${W}_{px}$ (kg_v_/kg_dry air_) is the humidity ratio of air evaluated at ${t}_{px}$; and $l(\mathrm{m})$ is the plate length.

#### Mass conservation

The water vapor transferred from the humid air stream partially deposits as frost on the cold surface and partially diffuses into the porous frost sublayers as follows:

4 $${{\int }_{x=0}^{\lambda }{h}_{v}\left({W}_{a}-{W}_{px}\right)dx={\int }_{x=0}^{\lambda }{\rho }_{fx}d{S}_{fx}}/{d\tau dx}+{\int }_{x=0}^{\lambda } {{S}_{fx}d{\rho }_{fx}}/{d\tau }dx,$$

where ${h}_{v}$(kg/m^2^s) is the average convective mass transfer coefficient, ${\rho }_{fx}$(kg/m^3^) is the local frost density, ${S}_{fx}$(m) is the local frost thickness, and $\tau (\mathrm{s})$ is time.

## Experimental tests

### Sample preparation and test method

#### Sample preparation

Natural fractured rock masses are the most suitable test samples. However, the fractures in these samples are often irregularly developed, and the rock matrix is anisotropic. Numerous conditions can affect the test results, making it difficult to identify the main influencing factors. Therefore, cement blocks were prefabricated and spliced into a sample with a vertical through fracture to simulate the simplest type of rock mass: a rock mass with a vertical through fracture. The cement blocks were made of P.O42.5 cement and water with w/c ratio of 0.5 and a block size of 12 $\times$ 5 $\times$ 30 cm (length $\times$ width $\times$ height), and were maintained for 28 days (T = 20 ± 2 °C, RH > 95%) after pouring. The specific parameters of the cement block is shown in Table 2. The porosity and tensile strength of cement blocks are close to those of coarse sandstone, which can approximate the ice formation phenomenon in the fractured rock of coarse sandstone.

**Table 2:** Physical and Mechanical properties of the cement block.

	Porosity (%)	Hydraulic conductivity (cm/s)	Compressive strength (MPa)	Tensile strength (MPa)
Cement block	14.3	2.5 × 10^–13^	27.5	5

The blocks were saturated by immersion for 7 days. After the blocks are saturated, there was negligible water migration within the blocks over the entire test process. Two cement blocks were spliced into a fractured rock simulation model with a vertical through fracture. The surface of the fracture is basically flat and smooth.

#### Test method

##### Water supply and moisture migration measurement

**Figure 2:**
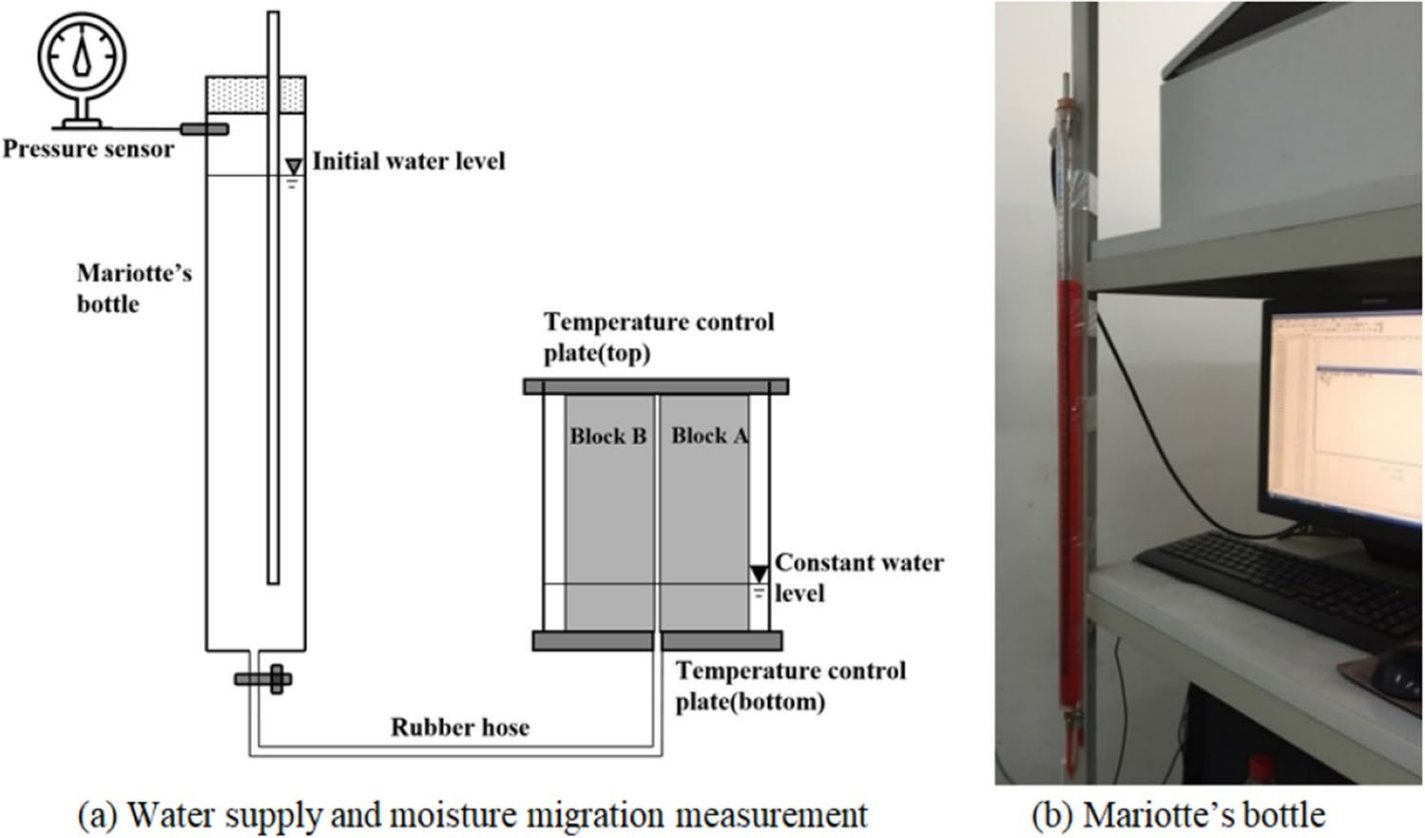
Schematic diagram of water supply and moisture migration measurement.

As shown in Fig. 2, a rubber hose is used to connect the Mariotte’s bottle to the bottom of the sample in order to maintain a constant water level. The bottle had an inner diameter of 2 cm and a height of 80 cm, with mm-scale accuracy; the water change volume per centimeter corresponded to 3.14 ml. A pressure sensor was connected to the top of the Mariotte’s bottle to measure the change in the water level. A red fluorescent dye was added to the water in the Mariotte’s bottle to visualize the migration path of the liquid water.

##### Temperature measurement

**Figure 3:**
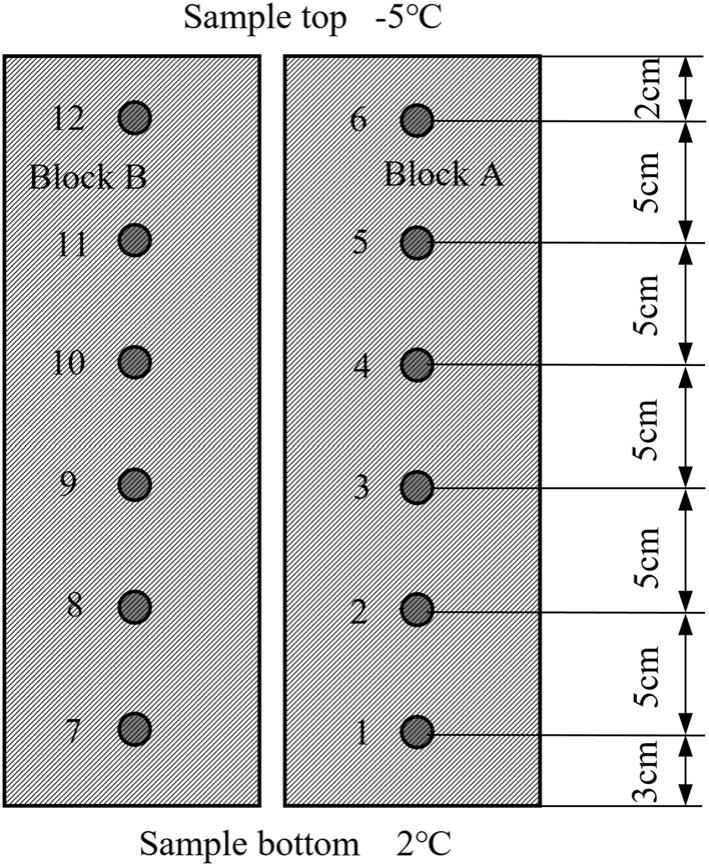
Temperature measurement (1–12 indicates the location and number of the temperature sensor).

A circular hole was prefabricated every 5 cm on the sides of the 5 cm $\times$ 30 cm test blocks. Each circular hole had a depth of 6 cm and a diameter of 8 mm. A temperature sensor was inserted into each hole of the sample and sealed with cement mortar. Platinum thermal resistance PT100 temperature sensors were used with a measurement accuracy of ± 0.3 °C. A row of temperature sensors were installed in blocks A and B to ensure the reliability of the measurement results, where the block positions and numbers are shown in Fig. 3.

##### Other equipment

The terminals of the temperature sensors and the pressure sensor were connected to an automatic data acquisition device (model number BGK-MICRO-40), whereby changes in the temperature and water level were automatically collected and recorded during the test. The study was conducted in a Xutemp freeze–thaw cycle box (Model Number XT5405FSC).

### Test procedure

**Figure 4:**
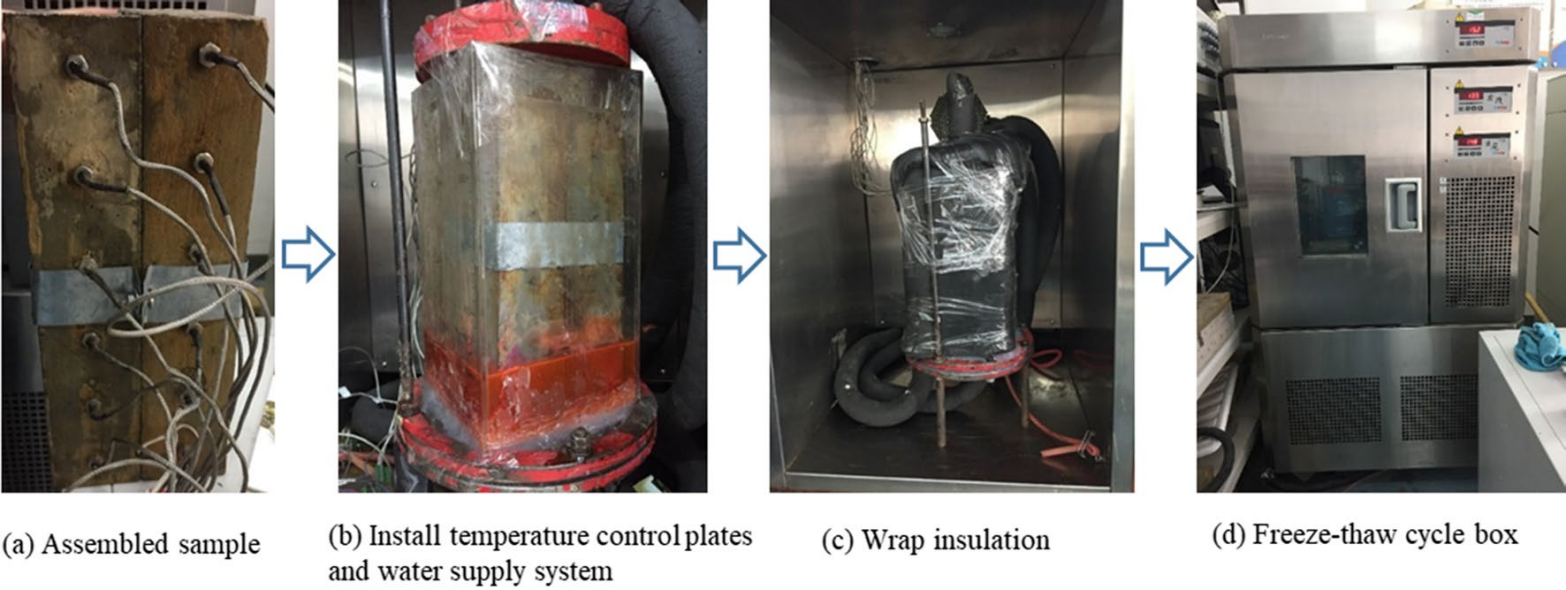
Test procedure.

The frost weathering test was conducted for a total of 260 h, during which the temperature in the sample and the water level in the Mariotte’s bottle were continuously monitored. The frost weathering test was carried out following 7 steps, as shown in Fig. 4. The specific process is described as follows.

- (1) Installation of temperature sensors: the PT100 temperature sensors were placed in the prefabricated holes of blocks A and B and sealed with cement mortar.
- (2) Saturation of test blocks: the blocks were saturated by immersion for 7 days.
- (3) Splicing of test block: the 12 cm $\times$ 30 cm faces of the two saturated blocks were spliced together and tightened with iron and bolts to form a sample with a vertical through fracture, as shown in Fig. 4a.
- (4) Sample installation: The sample was placed on the temperature control bottom plate in the freeze–thaw cycle box, the top plate was installed, and rubber sponge (thickness 20 mm) was used to wrap the rectangular plexiglass enclosure, the top and bottom plates, and the connection rubber hoses. Transparent tape was used to seal the gap between the top of the specimen and the plexiglass baffle, as shown in Fig. 4b,c.
- (5) Preparation of temperature and moisture migration measurement: the temperature sensors and the pressure sensor were connected to the automated data acquisition device. The Mariotte’s bottle was connected to the bottom of the sample using a rubber hose, the constant water level was 3 cm from the bottom plate, and the initial position of the liquid surface in the Mariotte’s bottle was 76 cm.
- (6) Initiation of temperature control system: in order to simulate the frost weathering effect of surface cooling on the internal bedrock, the top and bottom plate temperatures were set to − 5 °C and + 2 °C, respectively, and the temperature in the freeze–thaw cycle box was set to + 2 °C, as shown in Fig. 4d.
- (7) Date recording: the data acquisition system, including the temperature measuring elements and pressure sensors in the Mariotte’s bottle, was set to automatically collect data at 0.5–1 h time intervals.

## Test results and analysis

### Observed phenomenon after test

**Figure 5:**
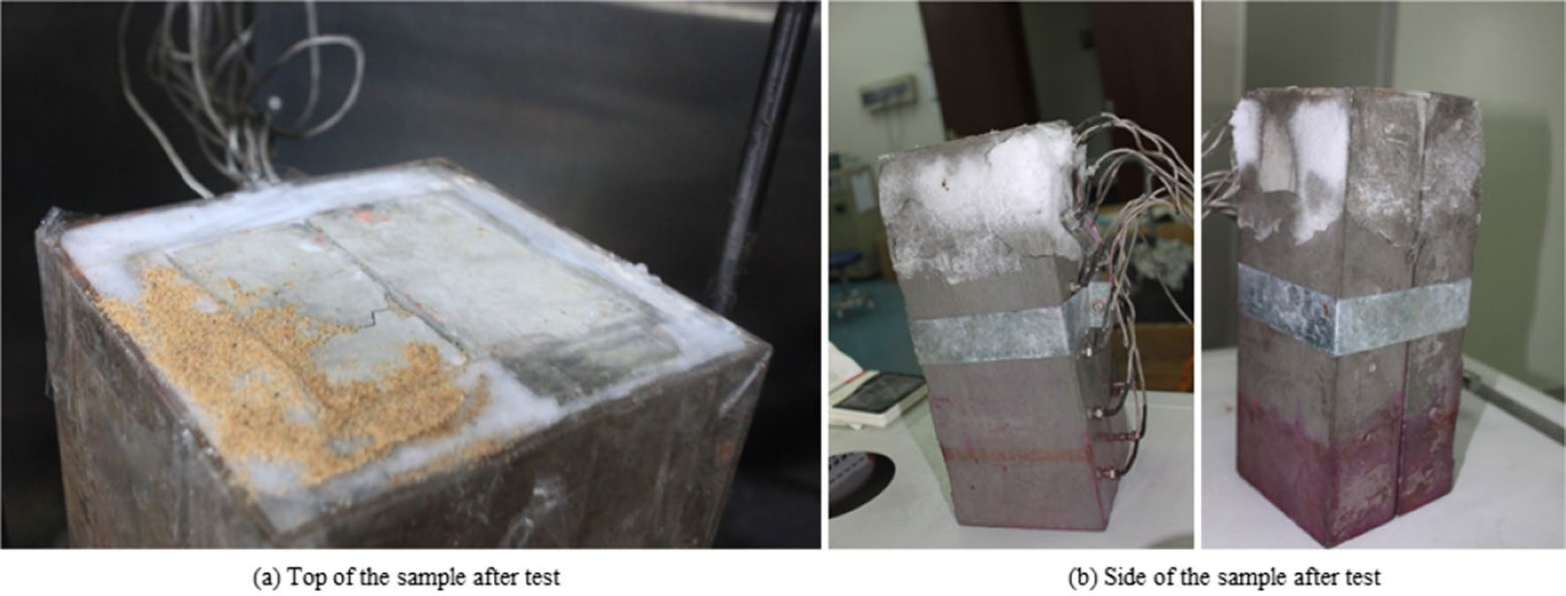
Sample appearance after test.

Removal of the temperature control top plate revealed approximately 5 ~ 10 mm of frost distributed on the inside of the tape used to seal the top of the sample, as shown in Fig. 5a. The sample was removed from the rectangular plexiglass enclosure, and a layer of hoarfrost with a thickness of approximately 3 to 7 mm was found to have condensed over the negative temperature range of the sample, as shown in Fig. 5b.

**Figure 6:**
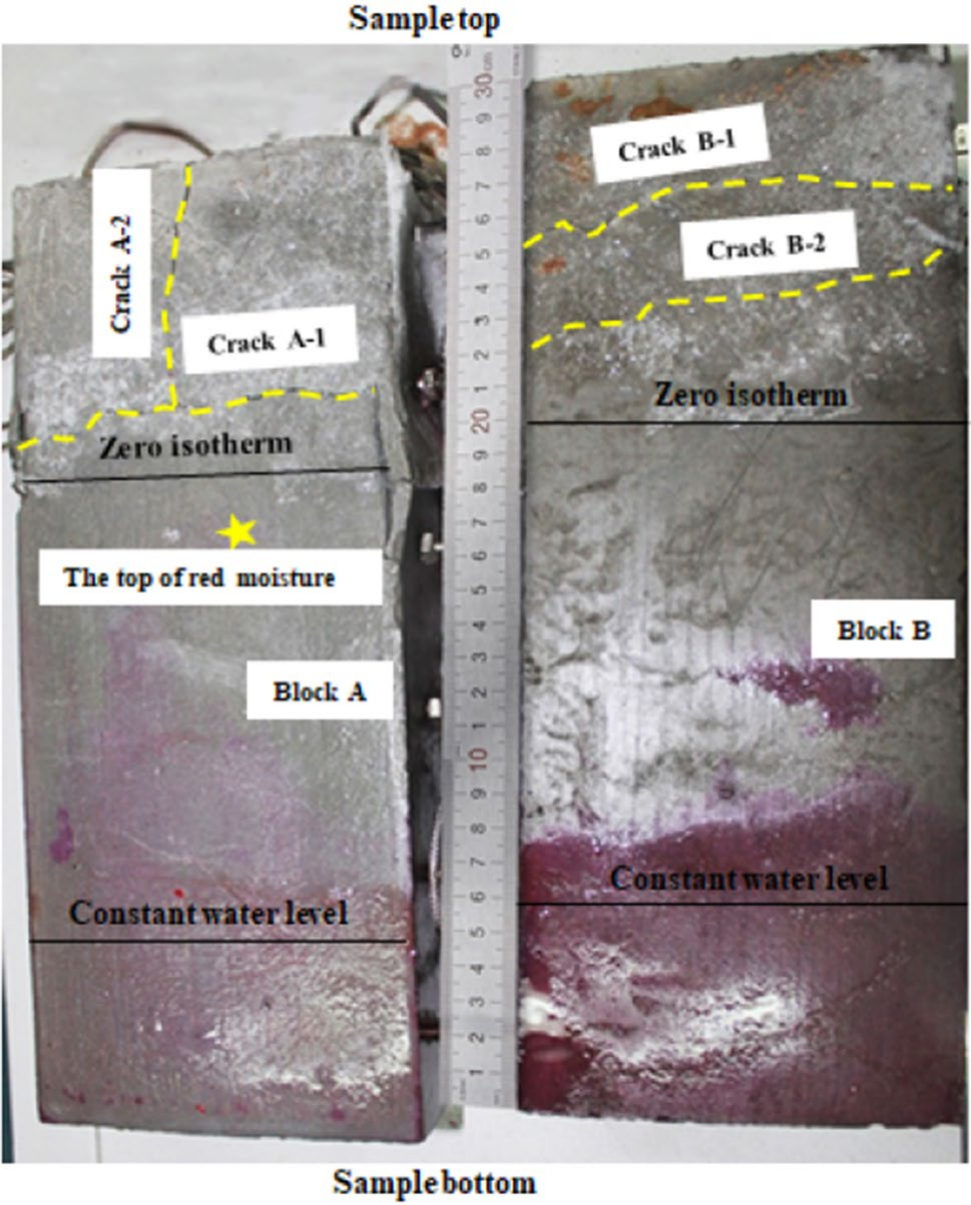
Prefabricated crack surface after test (color should be used).

As shown in Fig. 6, a level through crack A-1 and a vertical crack A-2 were generated over the negative temperature range on the surface of block A, and two nearly level through cracks B-1 and B-2 were generated over the negative temperature range on the surface of block B. Separation of blocks A and B revealed frost with a thickness of approximately 2 ~ 3 mm over the negative temperature range. There were distinct traces of water migration on the splicing surface, but the climb height was below the zero isotherm.

**Figure 7:**
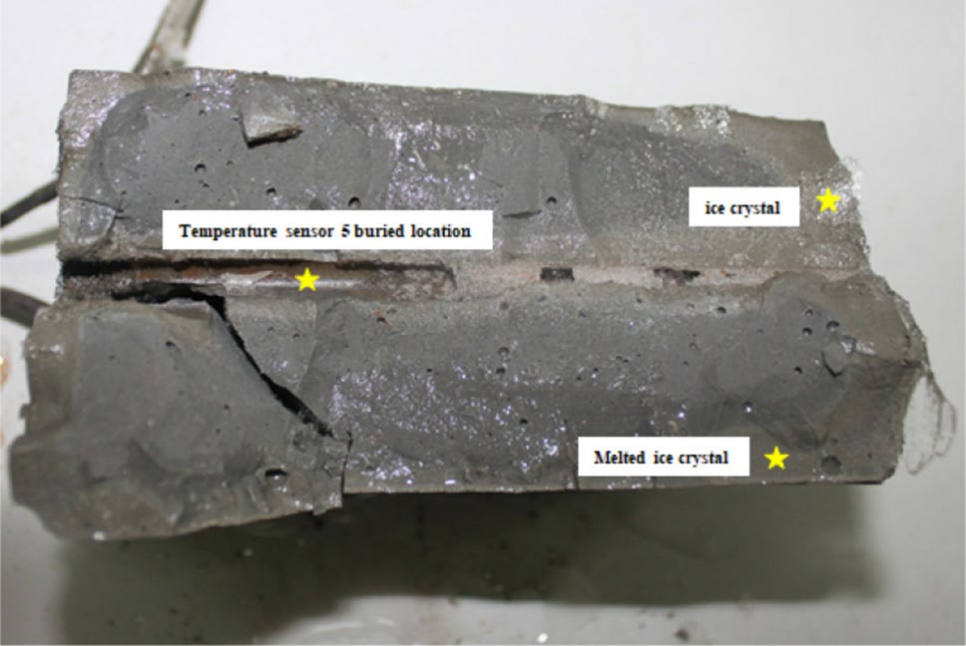
Surface of crack A-1 (color should be used).

Opening block A along cracks A-1 and A-2 revealed ice crystals on the surface, with some large pores on the crack surface, as shown in Fig. 7. Cracks A-1 and B-1 occurred where the temperature sensors were installed: the presence of the temperature measurement holes weakened the strength of the section, producing a weak interface that was relatively easy to crack.

### Temperature change in samples

The temperatures recorded by the temperature sensors were analyzed. Large data loss occurred during reading and recording at temperature sensors 1, 2, and 3, and dramatic and clearly unrealistic temperature changes were observed at temperature sensor 12. Therefore, temperature sensors 1, 2, 3 and 12 were excluded from the temperature analysis. The data obtained at temperature sensors 6, 11, 10, 9, 8 and 7 were used to represent the temperature changes in the sample from the top down to 2, 7, 12, 17, 22 and 27 cm, respectively.

**Figure 8:**
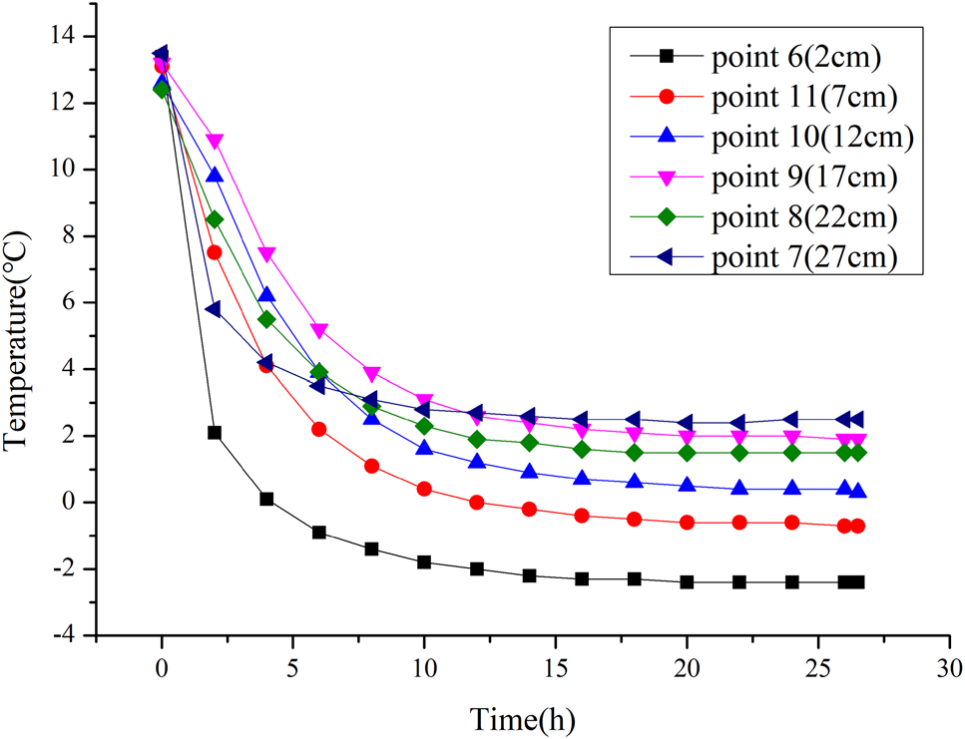
Temperature variation curve for different temperature measurement points inside sample (Note: the temperature does not change after the 18th hour).

Figure 8 shows a large slope for the temperature curve of each measurement point within the first 8 h, indicating a rapid drop in the temperature. The temperature change at each measurement point slows down from the 10th hour onwards and remains unchanged after 18 h, i.e. the nearly horizontal curve indicates that the heat exchange between the entire sample and the exterior has reached equilibrium and a stable temperature field has formed inside the sample.

Figure 8 also shows that after the sample reached the stable temperature field, the zero isotherm was located between measurement points 10 and 11, that is, the negative temperature zone approximately 10 cm from the top of the sample.

### Water change during test

**Figure 9:**
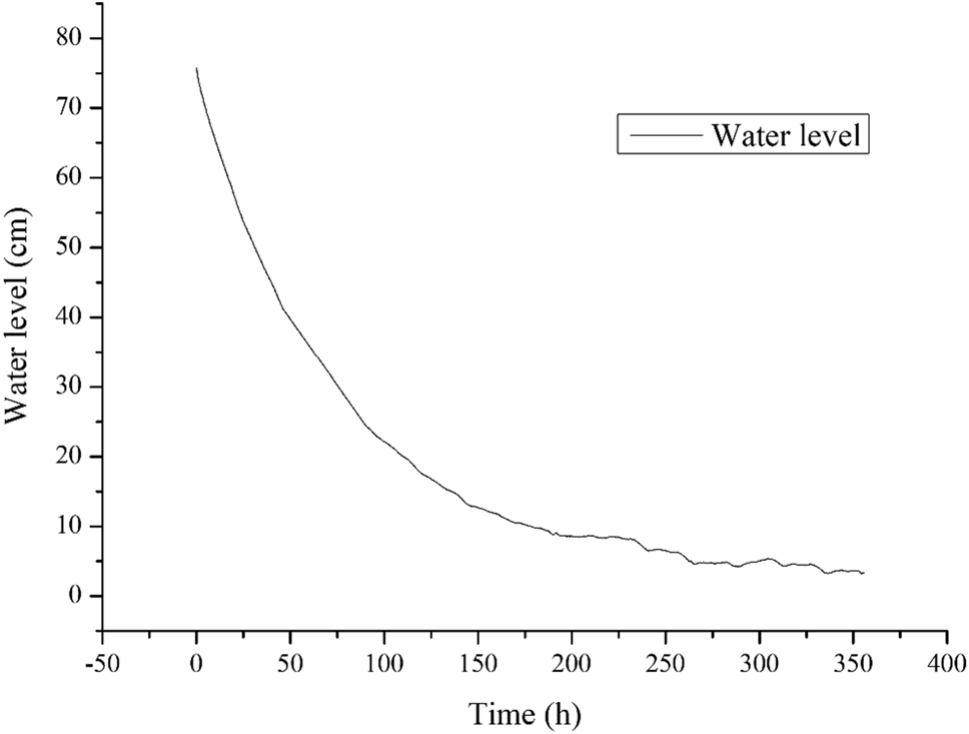
Water level in Mariotte’s bottle.

Figure 9 shows the change in the water level in the Mariotte’s bottle during the test.

In Fig. 9, the very large slope for the water level curve during the first 150 h indicates a rapid drop in the water level and fast water migration in the sample. The slope of the water level curve subsequently decreased up to 260 h and then gradually approached a horizontal line, indicating a relatively stable water level in the Mariotte’s bottle and that the water migration activity in the sample was stagnant or in a dynamic balance.

The corresponding water level at 260 h was 5.67 cm; considering the initial water level of 76 cm, the water migration during the test was (76−5.67)$\times$ 3.14 = 221 ml.

## Analysis and discussion

### The causes of cracks in cement blocks

As discussed in section “Experimental study of ice segregation and liquid water migration of rock”, for welded tuff (Akagawa and Fukuda^5^) and siliceous chalk (Murton et al.^8^) in the freezing test, it took a long time for a significant segregated ice layer to appear (> 1000 h), and for the relatively dense cement blocks, it should take even longer to produce the segregated ice layer, while this test lasted only 260 h. On the other hand, the segregated ice layer is generally perpendicular to the temperature gradient, while Crack-2 (Fig. 6) was in the same direction as the temperature gradient. In summary, the cracks on the cement blocks are most likely caused by in-situ volumetric expansion.

### Discussion of water migration patterns and pathways

Water migrated in vapor and liquid forms during the test. Water vapor condensed in the upper half of the sample to form frost at the top, as shown in Fig. 5. The main channels for water vapor migration were the vertical through fracture and the gap between the sample and the plexiglass baffle. Possible channels for liquid migration were the vertical through fracture and blocks A and B. In Fig. 6, the migration height of the liquid water in the vertical through fracture is very limited, at approximately 18 cm from the bottom of the sample, and has not yet reached the negative temperature zone. Considering that the space is completely filled with liquid water would only amount to 12 ml of water. Cement blocks A and B are saturated before the test starts, and from analysis in 4.1, it can be seen that the cracks on the cement blocks are most likely caused by in-situ volumetric expansion, thus, liquid water migration inside blocks A and B during the test period can be inferred to be very small.

In summary, a large quantity of water migrates in vapor form during the test. The main channels of migration are the prefabricated fracture and the gap between the sample and the plexiglass baffle.

### Novel mechanism of ice layer formation in fractured rock masses

The results presented in 3.1 ~ 3.3 and analysis in 4.1 ~ 4.2 lead to the following conclusions about the development of frost-heaving cracking in a fractured rock simulation model with a vertical through prefabricated fracture: (1) volumetric expansion produced a weak cross section in the negative temperature zone (because of pore development or the deterioration of the section strength by the prefabricated temperature measurement holes), resulting in initial cracks; (2) as the sample cooled, the water in the lower portion of the sample continuously migrated upward in vapor form to the negative temperature zone and condensed in the initial cracks; the subsequent expansion of the continuously growing ice crystals further expanded the crack; and (3) vapor migration stopped when the equilibrium condition was reached, and the ice crystals in the crack stopped growing.

**Figure 10:**
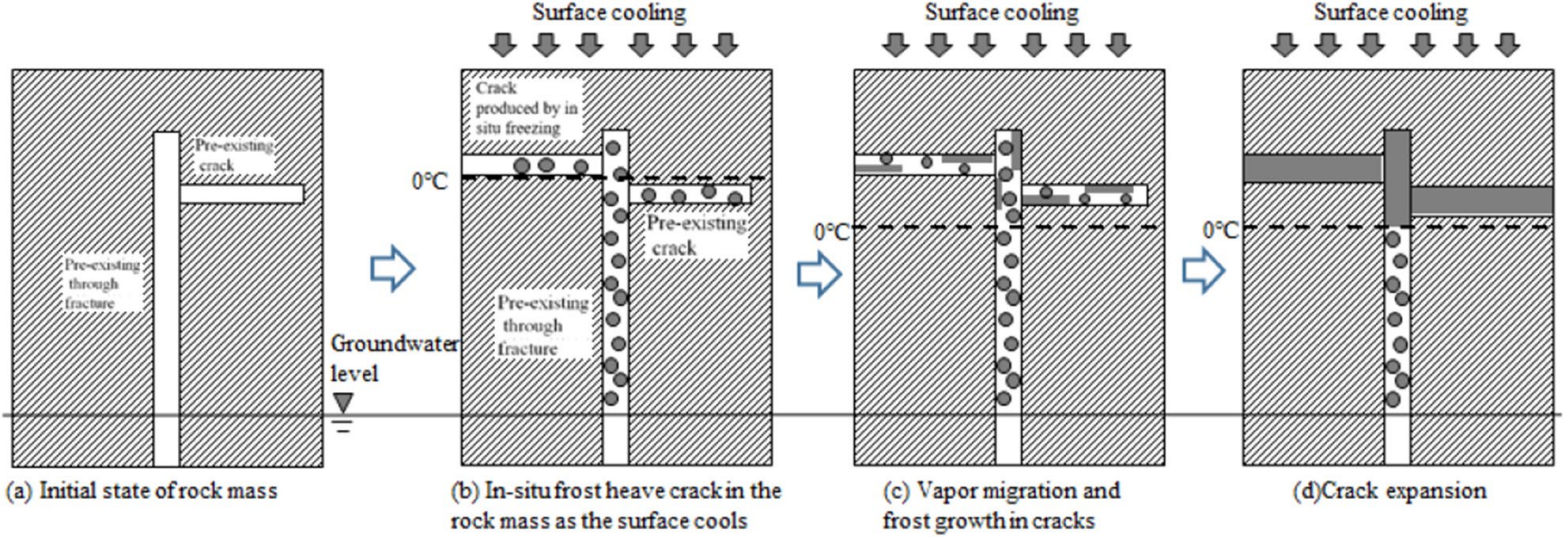
Formation of ice layer by initial crack-condensation effect.

Results from field research and the experiments presented above are used to propose a novel mechanism of ice layer formation in fractured rock masses, where the initial crack-frost effect can be described as follows: in rock masses with through fractures whose bottom is connected to the groundwater level, a drop in the surface temperature causes groundwater to migrate in vapor form through the fractures to the negative temperature zone and condense into frost in initial cracks (pre-existing or produced by in situ freezing). These frosts continuously accumulate and grow into an ice layer that expands the crack, resulting in frost-heaving damage to the rock mass. The entire process is shown in Fig. 10.

Thus, there are three conditions for ice layer formation in a fractured rock mass: the positive temperature zone is replenished by water, the penetrating fractures serve as water vapor migration channels, and the negative temperature zone contains cracks (that are preexisting or generated during frost heave). These conditions are relatively common in bedrock in cold regions, which supports the initial crack-frost effect as a mechanism for ice layer formation in a fractured rock mass.

### Driving force for water vapor migration

Assume that the water vapor in the rectangular plexiglass enclosure is in steady flow. Calculate the vapor migration due to the temperature gradient according to Eq. (2) in section “Vapor migration”.

For vapor migration in a rectangular plexiglass enclosure, the relation D_v_ = D_0_ holds approximately, where D_0_ is the free air diffusion coefficient, with a value of 10^-7^m^2^/s; considering that the temperature in the freeze–thaw cycle chamber is 2 °C, the saturated vapor density is 6.5 g/m^3^; $L$ is the latent heat of vaporization of water and is approximately equal to 2.50 kJ/g at 2 °C; the molar mass of water vapor ${\omega }_{w}$ is 18.016 g/mol;$\nabla T$ = (2− (− 5))/0.3 = 7 K/0.3 m; the gas constant R = 8.31 J/(mol·K); and T is 275 K; then the vapor flux caused by the temperature gradient is $-33.45\times {10}^{-7} \text{g}\cdot {\text{m}}^{-2}\cdot {\text{s}}^{-1}$. After 260 h under the test conditions, the flow section size is (0.15 $\times$ 0.15−0.12 $\times$ 0.1) = 0.0105 m^2^, such that the total vapor migration induced by the temperature gradient is $\left(33.45\times {10}^{-7}\mathrm{g}\cdot {\mathrm{m}}^{-2}\cdot {\mathrm{s}}^{-1}\right)\times 0.0105\; { \mathrm{m}}^{2}\times \left(260\times 3600\right)\; \mathrm{s}=328723\times {10}^{-7}\; \mathrm{g}$, which can be converted to volume of 0.0329 ml. Thus, the vapor migration induced by the temperature gradient is negligible compared to the overall migration of 221 ml recorded for the Mariotte’s bottle.

Therefore, the RH gradient is the main driving force for vapor migration in the rectangular plexiglass enclosure. In the approximately closed environment, the condensation of supersaturated water vapor into frost at the top of the sample where it was cold caused the RH of the air at the top of the sample to drop; thus, water vapor continuously migrated from the bottom to the top under the vapor pressure gradient, until the RH of the air at the top of the sample reached 100% again. The frost layer on the cold wall became increasingly thick during this cycle. The water vapor remained in a dynamic equilibrium until the frost layer stopped growing and then ceased migrating.

### Frosting mechanism at cold wall surface

The formation process of frost layer on the wall in the negative temperature region of the specimen is similar to the frosting process on the cold surface of thermodynamic studies, as shown in section “Frost mechanism on cold surface”. However, the growth of the frost layer in a crack is somewhat different from that on a general cold surface. The growth of the frost layer in the fracture occurs in a semiclosed environment. Supersaturated vapor condenses on the upper and lower rock walls at cold temperatures, and the growth of the frost layer on the upper walls affects that on the lower walls and vice versa; in addition, the frost layer grows on any ice present in the initial crack; finally, the growth and expansion of the frost layer in the fracture is always affected by the overlying load. Therefore, the growth of frost layers in fractures needs further study based on the above conclusions.

## Conclusion

The formation mechanism of an ice layer in a fractured rock mass was investigated by splicing two prefabricated blocks into a sample with a vertical through fracture and a constant water level at the bottom. A temperature control plate was placed at the top of the sample to simulate the unidirectional cooling of a rock mass. The following conclusions were drawn from the experimental results and a correlation analysis.

- (1) A novel mechanism for ice layer formation in a fractured rock mass is proposed in terms of an initial crack-frost effect: in a rock mass with through fractures with groundwater at the bottom, a drop in the surface temperature causes water to migrate, mainly in gaseous form, through the fractures to the negative temperature zone and condense into frost in initial cracks (which may be preexisting or generated during frost heave). These frosts continuously accumulate and grow into an ice layer that expands the crack, inflicting frost-heaving damage to the rock mass. The mechanism of the initial crack-frost effect may be more applicable to the formation of ice layers in fractured rock masses than the mechanisms reported in the literature. This effect does not require specific environmental conditions unlike volumetric expansion and ice segregation: the conditions required for the initial crack-frost effect are through fractures with groundwater at the bottom and initial cracks in the negative temperature zone.
- (2) Moisture mainly migrates in vapor form in through fractures. Water in rock fractures migrates in both liquid and vapor forms, where liquid water migration is driven by capillary suction but has a very limited climb height. The vast majority of water migrates in vapor form to condense into frost in cracks in the negative temperature zone.
- (3) Relative humidity (RH) gradients are the main driving force for water vapor migration. Supersaturated water vapor condenses into frost at the top of the sample where it is cold, and the drop in the RH in the air at the top of the sample creates a vapor pressure gradient that drives the continuous migration of water vapor from the bottom to the top of the sample. Temperature gradients have a very limited effect on water vapor migration.
- (4) The formation process of frost layer on the rock wall in the negative temperature region is similar to the frosting process on the cold surface of thermodynamic studies, therefore, this can be described by referring to the mechanism of frost on cold surfaces. However, the frosting mechanism in cracks is somewhat different from this and needs further study.
- (5) The late stage of frost growth in cracks involves the complex process of frost-ice transformation, and many microscopic tests and theoretical studies are required to elucidate this mechanism.

## Data Availability

The data used to support the findings of this research are included within the paper.
